# Financial burden and physical and emotional quality of life in COPD, heart failure, and kidney failure

**DOI:** 10.1371/journal.pone.0306620

**Published:** 2024-07-05

**Authors:** Seowoo Kim, Laura M. Perry, Brenna Mossman, Addison Dunn, Michael Hoerger

**Affiliations:** 1 Department of Psychology, Tulane University, New Orleans, Louisiana, United States of America; 2 Department of Medical Social Sciences, Northwestern University Feinberg School of Medicine, Chicago, Illinois, United States of America; 3 Center for Health Outcomes, Implementation, and Community-Engaged Science, Tulane University School of Medicine, New Orleans, Louisiana, United States of America; 4 Department of Psychiatry and Medicine, Tulane Cancer Center, and A.B. Freeman School of Business, Tulane University, New Orleans, Louisiana, United States of America; 5 Department of Palliative and Supportive Medicine, University Medical Center, New Orleans, Louisiana, United States of America; Universitas 17 Agustus 1945 - Jakarta, INDONESIA

## Abstract

Patients with chronic and serious illnesses experience significant quality of life concerns. More research is needed to understand the impact of financial burden on patients with COPD, heart failure, and kidney failure. Patients with COPD, heart failure, or kidney failure completed a cross-sectional online survey using validated measures of financial burden (general financial strain as well as financial toxicity attributable to treatment), physical quality of life (symptom burden and perceived health), and emotional quality of life (anxiety, depression, and suicidal ideation). ANCOVA was used to examine whether financial strain and financial toxicity were associated with physical and emotional quality of life, while accounting for key covariates. Among 225 participants with COPD (n = 137), heart failure (n = 48), or kidney failure (n = 40), 62.2% reported general financial strain, with 34.7% experiencing financial toxicity attributable to treatments. Additionally, 68.9% rated their health as fair or poor, experiencing significant symptom burden including fatigue, dyspnea, and chest pain. Participants also reported clinically relevant levels of anxiety (55.1%), depression (52.0%), and suicidal ideation (21.8%). In the total sample, financial strain was associated with worse physical and emotional quality of life on all measures (all Ps < .001). Financial toxicity attributable to treatment was not associated with quality of life in the total sample or subsamples. Patients with COPD, heart failure, and kidney failure face significant financial, physical, and emotional burdens. Financial strain appears to undermine physical and emotional quality of life. Our study highlights the demand for interventions aimed at mitigating financial strain and toxicity experienced by individuals with chronic illnesses.

## Introduction

Patients with serious chronic illnesses often experience high symptom burden and poor quality of life throughout their illness trajectory, which may last for months or years [1–3]. This burden is particularly pronounced in chronic obstructive pulmonary disease (COPD), heart failure, and kidney failure, three increasingly prevalent chronic organ failure diseases [4–6]. These conditions can cause a wide range of symptoms, such as pain, fatigue, loss of appetite, dyspnea, anxiety, and depression [1, 2, 7]. These symptoms can be debilitating and difficult to control, making it challenging for individuals to perform daily activities and maintain independence. Accordingly, many patients with COPD, heart failure, and kidney failure report decreased physical and emotional quality of life [8, 9].

Financial burden may diminish quality of life. Studies of financial burden in medical populations often focus on the general notion of “financial strain,” whether a family has the economic resources to meet their needs, or the narrower construct of “financial toxicity,” which focuses on the financial costs of medical care specifically. For example, chronic illnesses often require frequent medical visits, medications, imaging, tests, or even long-term care to manage the condition and mitigate symptoms [10]. The high cost of care can burden individuals and their families, causing financial toxicity [11–13]. Chronic conditions can also significantly impact a person’s financial stability due to lost income, debt, and added financial burdens associated with the condition, which can lead to broader financial strain [12, 14]. Previous research has shown that the financial burden resulting from medical care is significantly associated with reduced physical and emotional quality of life in cancer patients [15–18]. Studies have also shown that individuals with chronic illnesses experiencing financial burden are more likely to forgo or delay necessary medical care, which is associated with reduced physical quality of life and earlier mortality [19–21]. However, this evidence primarily focuses on patients with cancer and general financial burden, leaving knowledge gaps and uncertainties regarding the prevalence and impact of financial strain and toxicity on patients with non-cancer chronic illnesses and their quality of life. Furthermore, to the best of our knowledge, no prior research has examined the relationship between varied assessments of financial burden and both physical and emotional quality of life in chronic, non-cancer patients.

In this paper, we examined the associations between financial burden and physical and emotional quality of life among patients with non-cancer serious illnesses, specifically COPD, heart failure, and kidney failure. These three diagnoses were chosen based on the aim of the initial study that created the dataset used in the present analyses, which was to validate a novel scale that measures patient attitudes towards palliative care [22]. The study focused on these three diagnoses because they provide examples of relatively common non-malignant populations that have a demonstrated palliative care need. The present analyses included both general financial strain and financial toxicity and incorporated key indicators of physical quality of life (perceived health and symptom burden) and emotional quality of life (anxiety, depression, and suicidal ideation) that prior research has suggested may be sensitive to financial burden [15, 18, 19, 23, 24]. We hypothesized that patients who experience financial burden would be more likely to have worse physical and emotional quality of life.

## Methods

### Overview

The study involves a comprehensive analysis of existing data from the Palliative Care Attitudes Scale (PCAS-9) development study [22], focusing on patients with COPD, heart failure, and kidney failure. Participants completed a cross-sectional online survey, responding to validated measures that assessed financial burden, physical quality of life, and emotional quality of life. The primary goal was to examine associations between financial burden and both physical and emotional quality of life, employing statistical methods such as ANCOVAs and subgroup analyses to explore the effects within each illness-defined group.

### Participants and procedures

This is a primary analysis of existing data from the Palliative Care Attitudes Scale (PCAS-9) development study [22], which involved recruiting patients with serious illnesses to complete an online cross-sectional survey. Participants with a history of COPD, heart failure, and kidney failure were recruited from February 21, 2018, to March 27, 2018, using the National Institutes of Health (NIH)-sponsored ResearchMatch patient recruitment database and social media. Eligible participants were notified of the opportunity to participate if they were registered with ResearchMatch.org under one of the eligible health conditions (COPD, heart failure, kidney failure), or if they belonged to an online support group on Facebook, a supportive care website, or another social media page dedicated to one of the eligible health conditions. Participants were included if they had been diagnosed by a pulmonologist (COPD), cardiologist (heart failure), or other doctor within the last 12 months with one of the following diagnoses: a) COPD, chronic bronchitis, emphysema, or chronic lower respiratory disease; b) heart failure or congestive heart failure (CHF); c) kidney failure or end-stage kidney disease. Individuals who indicated they had heart failure also self-reported symptoms based on the Framingham Heart Failure criteria [25], which was used as an additional criterion for inclusion in this subgroup. All participants were required to be at least 18 years old and able to read English. Participants provided written informed consent prior to the survey. The study was approved by the Tulane University Institutional Review Board (#2017–723).

### Measures

#### Participant characteristics

Participants self-reported their age, education, gender, race, ethnicity, geographic location, and marital status. They also provided their health information including the recency of diagnosis, disease-specific treatments received, and presence of comorbidities.

#### Financial burden

Financial strain was assessed using a four-item measure that has been previously used in a large sample of older adults in the U.S. [26] and a sample of adults with cancer [27]. The items asked participants whether their income was enough for: 1) food and housing, 2) clothing, medicine, home repairs, and transportation, 3) going out for a meal and entertainment, and 4) a week-long vacation if health allowed. Participants indicating inability to afford one or more options were classified as financially strained.

Financial toxicity was assessed using a financial domain item from the Patient Satisfaction Questionnaire [28]. The item stated, “I have to pay for more of my medical care than I can afford,” and participants responded on a 5-point scale from 1 (strongly disagree) to 5 (strongly agree). Participants who rated the item 4 (agree) or 5 (strongly agree) were classified as experiencing financial toxicity.

#### Physical quality of life

Participants in all three diagnostic samples completed the SF-1 perceived health item, assessing their physical quality of life. The item asked, “In general, how would you say your health is?” Participants responded on a 5-point scale from 1 (poor) to 5 (excellent) [29]. Additionally, participants completed assessments of symptom burden tailored to their specific diagnosis. Participants with a history of COPD completed the Functional Assessment of Chronic Illness Therapy-Dyspnea (FACIT-Dyspnea; α = .92) ten-item short form developed and validated specifically for participants with COPD [30]. Participants with a history of heart failure completed six items from the Kansas City Cardiomyopathy Questionnaire (KCCQ; α = .86) assessing heart failure symptom burden and frequency [31]. Participants with a history of kidney failure completed the six-item short form of the Kidney Disease Quality of Life (KDQOL; α = .79) instrument with items assessing the burden, symptoms, and physical effects of kidney disease [32]. Higher scores on these measures indicated worse quality of life.

#### Emotional quality of life

Emotional quality of life was assessed using two Patient-Reported Outcomes Measurement Information System (PROMIS) short-forms assessing anxiety (four items; α = .86) and depression (four items; α = .96) [33, 34]. Raw scores were converted into T-scores in line with current PROMIS reporting standards [35]. A cut score of ≥55 was used to identify individuals with clinically relevant symptoms of anxiety or depression [36]. The PROMIS depression and anxiety scales have shown evidence of validity among similar samples, including individuals with chronic obstructive pulmonary disease (COPD) and heart failure [37]. Participants also completed item 9 of the Patient Health Questionnaire (PHQ-9) to detect suicidal ideation [38]. The item asked, “I had thoughts that I would be better off dead, or of hurting myself in some way,” and participants responded on a 4-point scale from 0 (not at all) to 3 (most of the time). Participants who rated the item a 1 (some of the time), 2 (a good part of the time), or 3 (most of the time) were classified as having suicidal ideation.

### Statistical analysis

We conducted pooled analyses across all participants and subgroup analyses for each illness-defined group. Pooled analyses for SF-1 and emotional quality of life measures combined responses across sample, as all participants completed these same measures. Pooled analyses of symptom burden standardized scores within each subsample using each group’s disease-specific symptom burden measure (KDQOL, KCCQ, FACIT-Dyspnea) and then T-scored them based on pooled means and standard deviations. We obtained descriptive statistics to provide a summary of the demographic, health, financial, and quality of life measures for each illness subgroup and the total sample. Then, zero-order associations between demographic variables and financial burden were examined using chi-squared tests of independence.

For primary hypothesis testing, we conducted analyses of covariance (ANCOVAs) for the total sample to identify the associations between financial burden (financial strain, financial toxicity) and physical (SF-1, symptom burden) and emotional (PROMIS anxiety, PROMIS depression, PHQ suicide) quality of life outcome. Exploratory subgroup analyses analyzed the impact of financial strain within each disease group; separate ANCOVA models employed each disease-specific quality-of-life measure as a dependent variable. Covariates included age, gender, marital status, educational level, and recency of diagnosis, which are potential confounders identified as relevant to quality of life in other studies [39, 40]. The Benjamini-Hochberg correction method was used to control for false discovery rate (*Q* = .25). The standardized mean differences, Cohen’s *d*, were used to calculate effect sizes from covariate-adjusted means and standard deviations from the ANCOVAs.

## Results

### Demographics

The sample included 225 total participants (Table 1), including people with a history of COPD (n = 137; 60.9%), heart failure (n = 48; 21.3%), and kidney failure (n = 40; 17.8%). Participant’s age ranged from 23 to 87 years, with an average of 61.21 years. Among COPD patients, the median time since diagnosis was 9 years, with 8.0% in the past year. Their treatment regimens included oxygen therapy (56.2%), pulmonary rehabilitation (46.0%), and lung transplant surgery (14.6%). For heart failure patients, the median time since diagnosis was 3.70 years, with 29.2% diagnosed in the past year. Treatment regimens for heart failure patients included implantable cardiac defibrillators (35.4%), angioplasty surgery (27.1%), and pacemaker or cardiac resynchronization therapy (25.0%). For kidney failure patients, the median time since diagnosis was 3.75 years, with 27.5% diagnosed in the past year. Treatment regimens for kidney failure patients included dialysis (80.0%) and kidney transplant surgery (60.0%).

**Table 1:** Participant characteristics.

Characteristic	Total	COPD	Heart Failure	Kidney Failure
	(N = 225)	(n = 137)	(n = 48)	(n = 40)
Age, yrs	61.21 ± 12.53	65.45 ± 11.33	55.94 ± 11.10	53.03 ± 11.83
Gender, female	141 (62.7%)	81 (59.1%)	31 (64.6%)	29 (72.5%)
Education, bachelor’s or higher	94 (41.8%)	56 (40.9%)	19 (39.6%)	19 (47.5%)
Relationship status, married	106 (47.1%)	64 (46.7%)	20 (41.7%)	22 (55.0%)
Recent diagnosis, past year	36 (16.0%)	11 (8.0%)	14 (29.2%)	11 (27.5%)
Financial strain, present	140 (62.2%)	84 (61.3%)	34 (70.8%)	22 (55.0%)
Financial toxicity, present	78 (34.7%)	48 (35.0%)	19 (39.6%)	11 (27.5%)
Perceived health				
Excellent	5 (2.2%)	4 (2.9%)	0 (0.0%)	1 (2.5%)
Very good	12 (5.3%)	8 (5.8%)	2 (4.2%)	2 (5.0%)
Good	53 (23.6%)	41 (29.9%)	4 (8.3%)	8 (20.0%)
Fair	98 (43.6%)	53 (38.7%)	22 (45.8%)	23 (57.5%)
Poor	57 (25.3%)	31 (22.6%)	20 (41.7%)	6 (15.0%)
Symptom burden, mean	50.00 ± 10.00	14.54 ± 8.35	21.02 ± 7.93	14.03 ± 4.79
Clinically relevant anxiety, present	124 (55.1%)	66 (48.2%)	39 (81.3%)	19 (47.5%)
Clinically relevant depression, present	118 (52.4%)	63 (46.0%)	33 (68.8%)	22 (55.0%)
Suicidal ideation, present	49 (21.8%)	20 (14.6%)	16 (33.3%)	13 (32.5%)

*Note*. For symptom burden, each subgroup completed a disease-specific measure; scores were standardized across groups and put on the T-scale (M = 50, SD = 10).

### Financial burden

Financial strain was present in 62.2% of the total sample, 61.3% of the COPD group, 70.8% of the heart failure group, and 55.0% of the kidney failure group. Table 2 shows that participants who were younger (*P* = 0.016), unmarried (*P*< 0.001), more recently diagnosed (*P* = 0.036), or without a bachelor’s degree (*P*< 0.001) were more likely to experience financial strain.

**Table 2:** Point prevalence of financial burden within demographic and health subgroups.

Characteristic	Financial Strain		*P*-value	Financial Toxicity		*P*-value
	Present	Absent		Present	Absent	
	(N = 140)	(N = 85)		(N = 78)	(N = 147)	
	n (%)	n (%)		n (%)	n (%)	
**Age, yrs**						
<65	92 (68.7%)	42 (31.3%)	0.016	54 (40.3%)	80 (59.7%)	0.031
≥65	48 (52.7%)	43 (47.3%)		24 (26.4%)	67 (73.6%)	
**Gender**						
Male	49 (58.3%)	35 (41.7%)	0.353	22 (26.2%)	62 (73.8%)	0.039
Female	91 (64.5%)	50 (35.5%)		56 (39.7%)	85 (60.3%)	
**Marital Status**						
Unmarried	88 (73.9%)	31 (26.1%)	<0.001	51 (42.9%)	68 (57.1%)	0.006
Married	52 (49.1%)	54 (50.9%)		27 (25.5%)	79 (74.5%)	
**Education**						
Bachelor’s absent	94 (71.8%)	37 (28.2%)	<0.001	56 (42.7%)	75 (57.3%)	0.003
Bachelor’s present	46 (48.9%)	48 (51.1%)		22 (23.4%)	72 (76.6%)	
**Time since diagnosis**						
≤ 1 year	28 (77.8%)	8 (22.2%)	0.036	15 (41.7%)	21 (58.3%)	0.336
>1 year	112 (59.3%)	77 (40.7%)		63 (33.3%)	126 (66.7%)	

*Note*. *N* = 225. P-values were obtained from chi-squared tests of independence to examine the zero-order association between each participant characteristic (dichotomous variable) and presence of financial strain and financial toxicity (dichotomous variables).

Financial toxicity, which is financial burden specifically related to the cost of treatment, was present in 34.7% of the total sample, 35.0% of the COPD group, 39.6% of the heart failure group, and 27.5% of the kidney failure group. Table 2 shows that participants who were younger (*P* = 0.031), female (*P* = 0.039), unmarried (*P* = 0.006), or without a bachelor’s degree (*P* = 0.003) were more likely to experience financial toxicity.

### Quality of life

As shown in Table 1, most participants rated their physical health as either poor or fair (n = 155; 68.9%), varying by subgroup: COPD (61.3%), heart failure (87.5%), and kidney failure (72.5%). The measure of symptom burden varied within each subgroup showing substantial variability. For emotional quality of life, approximately half of the total sample experienced clinically relevant levels of anxiety (55.1%) and depression (52.4%), with 21.8% experiencing suicidal ideation. The subgroup with the highest prevalence of emotional symptoms was heart failure (81.3% for clinically relevant anxiety, 68.8% for clinically relevant depression, and 33.3% for suicidal ideation).

### Associations between financial burden and quality of life

#### Physical quality of life

Table 3 shows that across all three diagnostic groups, patients experiencing financial strain had worse physical and emotional quality of life while accounting for key demographics and health covariates including age, gender, education level, marital status, and recency of diagnosis. In the total sample, financial strain was associated with worse perceived health (*d* = 0.71, *P*< 0.001) and greater symptom burden (*d* = 0.55, *P*< 0.001), with effects varying by subgroup. Financial toxicity was not associated with perceived health status or symptom burden in the total sample and subsamples.

**Table 3:** Financial strain and quality of life in chronic disease.

Quality of Life Measures	Financial Strain		Standardized Mean Difference (Cohen’s d)	*P*-value
	Present	Absent		
	M (SD)	M (SD)		
**Total Sample (*N* = 225)**	*n* = 140	*n* = 85		
Physical				
SF-1 perceived health	1.91 (0.91)	2.56 (0.93)	0.71	<0.001*
Symptom burden	52.03 (9.71)	46.66 (9.93)	0.55	<0.001*
Emotional				
PROMIS Anxiety	57.47 (10.21)	52.33 (10.46)	0.50	<0.001*
PROMIS Depression	57.00 (10.05)	51.11 (10.27)	0.58	<0.001*
PHQ Suicide	0.40 (0.60)	0.10 (0.62)	0.49	<0.001*
**COPD** (*N* = 137)	*n* = 84	*n* = 53		
Physical				
SF-1 perceived health	2.05 (0.96)	2.64 (0.98)	0.61	0.001*
Symptom burden	15.62 (9.60)	12.81 (9.91)	0.29	0.121
Emotional				
PROMIS Anxiety	55.52 (9.67)	52.10 (9.90)	0.35	0.044*
PROMIS Depression	55.40 (9.18)	51.25 (9.40)	0.45	0.020*
PHQ Suicide	0.23 (0.44)	0.07 (0.45)	0.36	0.060
**Heart Failure** (*N* = 48)	*n* = 34	*n* = 14		
Physical				
SF-1 perceived health	1.50 (0.75)	2.37 (0.79)	1.13	0.002*
Symptom burden	22.68 (8.11)	16.99 (8.58)	0.68	0.050
Emotional				
PROMIS Anxiety	63.57 (9.52)	54.69 (10.07)	0.91	0.017*
PROMIS Depression	60.62 (10.86)	50.72 (11.48)	0.89	0.006*
PHQ Suicide	0.71 (0.80)	0.00 (0.85)	0.86	0.015*
**Kidney Failure** (*N* = 40)	*n* = 22	*n* = 18		
Physical				
SF-1 perceived health	1.93 (0.88)	2.58 (0.89)	0.79	0.029*
Symptom burden	16.22 (4.36)	11.35 (4.43)	1.11	0.003*
Emotional				
PROMIS Anxiety	57.41 (12.18)	48.78 (12.38)	0.70	0.048
PROMIS Depression	59.03 (12.83)	49.14 (13.04)	0.76	0.041*
PHQ Suicide	0.64 (0.88)	0.22 (0.89)	0.47	0.183

*Note*. For Cohen’s d, the effect size indicates the standardized mean difference, with positive values indicating better quality of life among people with lower financial strain. For symptom burden, an averaged composite T-score of the three disease-specific symptom burden measures (KDQOL, KCCQ, FACIT-Dyspnea) was used.

*Statistically remained significant after application of the Benjamini-Hochberg adjustment at a false discovery rate of 0.25.

#### Emotional quality of life

Patients experiencing financial strain also had worse emotional quality of life while accounting for demographic and health covariates (Table 3). In the total sample, financial strain was associated with greater anxiety (*d* = 0.52, *P*< 0.001), depression (*d* = 0.62, *P*< 0.001), and suicidal ideation (*d* = 0.49, *P*< 0.001), with effects varying by subgroup. Financial toxicity was not associated with anxiety, depression, or suicidal ideation in either the total sample or the subsamples.

## Discussion

This research documents the financial, physical, and emotional burdens experienced by patients with COPD, heart failure, and kidney failure. Across all participants, 62% experienced financial strain in their lives, with 35% experiencing financial toxicity attributable to their treatments. Physically, 69% described having only fair or poor health, accompanied by significant physical symptom burden including fatigue, dyspnea, and chest pain. Emotionally, 55% had clinically relevant levels of anxiety, 52% depression, and 22% suicidal ideation. This finding is consistent with prior research that revealed a notable proportion of individuals with chronic illnesses reporting poor health and experiencing symptoms of emotional distress, including anxiety and depression [41–43]. Overall, patients who experienced more financial strain had worse physical and emotional quality of life. To the best of our knowledge, this is the first study to establish an association between financial strain and both physical and emotional quality of life in these three chronic nonmalignant disease groups. Findings highlight the importance of addressing financial burdens experienced by patients with chronic illnesses.

Our findings expand upon prior research primarily conducted in cancer, which identified financial burden as a risk factor for worse physical quality of life [17, 18], higher symptom burden [19, 27], and worse emotional quality of life [44, 45], indicating that the negative impact of financial burden on quality of life may generalize to populations with non-cancerous chronic illnesses. Furthermore, our findings build upon and contextualize prior literature documenting the financial burden of COPD, heart failure, and kidney failure. Two systematic reviews have highlighted that COPD is associated with higher healthcare resource use, hospital admissions, and treatment costs than other chronic respiratory diseases [11, 46]. Recent studies on heart failure medical costs showed that the annual median costs for heart failure are about $24,383 per patient, with average family out-of-pocket expenditures of about $4,423, leading to financial burden for patients and families [47, 48]. Although limited literature exists on the financial burden on kidney failure patients and their families, studies on chronic kidney disease and kidney stone patients have reported substantial medical costs [49, 50]. It was interesting to note that in our investigation, patients’ general financial strain was more closely associated with their quality of life, whereas in the cancer literature, financial toxicity directly attributable to treatment may play a more critical role [27, 51]. If forced to speculate, we would hypothesize that financial toxicity matters more in cancer due to the often-sudden diagnosis and condensed treatment window, whereas COPD, heart failure, and kidney failure may be characterized by more gradual financial declines as the illnesses progress over years or decades. Thus, findings generalize beyond the cancer literature to show the importance of financial burden for quality of life, with the caveat that such financial burdens may accumulate more insidiously in COPD, heart failure, and kidney failure than in cancer.

Several additional findings stood out. Age, marital status, and education status were significantly associated with both financial strain and toxicity. These results are consistent with previous studies, which suggest that older, married, and more educated individuals tend to experience less financial burden and emotional distress [27, 52–54]. This may be due to younger patients’ lack of financial resources for treatment, unmarried patients’ lower family incomes, and less educated patients’ reduced access to resources compared to their counterparts. Furthermore, our study found that gender was significantly associated with financial toxicity but not with financial strain. This suggests that women may struggle more with the financial burden of medical treatment due to their lower average income, potentially exacerbating the issue. Finally, we found that while recency of diagnosis was significantly associated with financial strain, it did not appear to have a significant impact on financial toxicity. It is possible that patients experience more general financial difficulties or have obligations unrelated to healthcare costs, possibly due to participants’ access to financial resources that mitigated the effects of financial toxicity, such as health insurance that covered a large portion of healthcare expenses.

### Study limitations

This study had strengths and limitations. Strengths included the use of financial measures underutilized in patient-oriented research as well as the use of well-validated measures of quality of life, including the innovative PROMIS measures. The study’s cross-sectional design, however, restricts its ability to establish firm causal relationships, and the reliance on self-reported data compounds this limitation. The internet sampling methodology may have biased patient selection towards those with higher financial burdens. Furthermore, the sample demonstrated significant symptom burden, partly due to the purpose of the parent study validating Palliative Care Attitudes Scale [22]. Because of this, our findings may not generalize to COPD or other chronically ill patients who experience fewer or better-managed symptoms. The majority (95.1%) of the sample resided in the US, where the healthcare system may impose higher costs on patients compared to other countries, potentially exacerbating financial burden and disparities [55]. Similar analyses will need to be replicated in other countries and healthcare systems to determine if the findings generalize beyond the US population. Lastly, the samples of heart failure and kidney failure participants were relatively small, potentially leading to underestimation of effects in inferential tests. We reported several descriptive analyses for disease subgroups and the total sample to accurately characterize the results. Future research could enhance our understanding of the impact of financial strain on quality of life by employing longitudinal designs across various healthcare systems. Such studies would allow analysis of how financial strain evolves over time and interacts with socio-economic and health-related factors to impact patient’s quality of life. Additionally, comparative analyses between cancer and non-cancer patients could aid in better understanding the differential impact of financial strain across patient populations in the future. Lastly, employing a singular, objective measure to assess symptom severity across various disease conditions could facilitate more effective comparison of symptoms among patients with different disease statuses. By integrating these approaches, researchers can better understand how financial strain impacts health outcomes and form strategies to alleviate its effects on individuals with chronic illnesses. Despite these limitations, the current study was unique in its use of multiple measures of financial burden and its assessment of physical and emotional quality of life in a non-cancerous chronically ill population.

### Clinical implications

Our study highlights the demand for interventions aimed at mitigating financial strain and toxicity experienced by individuals with chronic illnesses. It is crucial for healthcare providers to consider socioeconomic difficulties while planning and delivering medical services for this patient population. Regular screening for financial burdens during check-ups, referral to financial counseling or assistance programs, and communication with insurance companies to minimize out-of-pocket expenses can reduce financial burden and improve patients’ quality of life. In particular, psychosocial, supportive, and palliative care teams can help patients manage costs and navigate distress effectively. Early referral to these teams may help mitigate financial, physical, and emotional burdens.

At the policy level, healthcare systems and policymakers should address systemic issues that contribute to financial burdens for seriously ill patients. This may involve advocating for policies that provide greater access to affordable healthcare and insurance, reducing out-of-pocket costs, and expanding programs that enhance quality of life through increased access to multidisciplinary supportive care services.

## Conclusion

In conclusion, our study demonstrates that patients with non-cancerous chronic conditions who experience financial strain have poorer physical and emotional quality of life. These findings underscore the need for interventions aimed at reducing the financial burden of chronic conditions and enhancing quality of life for affected individuals.
